# The associations of fatty acids related dietary patterns with overweight and obesity among Chinese children

**DOI:** 10.1186/s41043-024-00549-9

**Published:** 2024-04-23

**Authors:** Zhi Huang, Keyu Ma, Xiaochen Yin, Ziming Li, Ming Chen, Yujie Duan, Li Li, Yuming Hu

**Affiliations:** 1 School of Public Health and Laboratory, Hunan University of Medicine, Jinxi Road No.492, 418000 Huaihua, China; 2 School of Public Health, University of South China, Changsheng West Road No.28, 421001 Hengyang, China; 3 The department of Toxicology, Hunan Provincial Center for Disease Control and Prevention, Furong Road No.450, 410005 Changsha, China

**Keywords:** Fatty acids, Dietary patterns, Overweight, Obesity, Children

## Abstract

**Background:**

Childhood overweight and obesity is becoming an emerging face of malnutrition. The aims of this study were to develop fatty acid (FAs) related dietary patterns and explored the associations of FAs related dietary patterns with overweight and obesity among Chinese children.

**Methods:**

An observational study was conducted on 435 children aged 4 to 7 years old in South Central China. Erythrocyte FAs composition was analyzed by gas chromatography-mass spectrometry. Diet was collected by food frequency questionnaires and dietary patterns were evaluated by reduced rank regression. The logistic regression analysis was used to exploring the association of dietary patterns with overweight and obesity.

**Results:**

The prevalence of overweight, obesity, and overweight or obesity were 6.52, 4.59, and 11.11% in Chinese children, respectively. Twenty five types of FAs were detected in erythrocyte of children and four FAs related dietary patterns were identified. The dietary pattern positively correlated with n-3 PUFAs, but negatively with SFAs,was characterized by high intake of fish, shrimp, crab and shellfish, leaf-off vegetable, nuts, and tubers, which have a significantly decreased overweight risk (OR = 0.580, 95%CI: 0.375 ∼ 0.895, *P* = 0.014).The pattern positively strong associated with n-6 PUFAs, but negatively strong with n-3 PUFAs, had high intake of snacks, leaf-off vegetable, fresh beans, and coarse cereals, which have a significantly decreased obesity risk (OR = 0.518, 95%CI: 0.325 ∼ 0.827, *P* = 0.006).

**Conclusion:**

Four FAs related dietary patterns were identified. The dietary pattern with high intake of fish, shrimp, crab and shellfish decreased overweight risk by increasing n-3 PUFAs, and decreasing SFAs. The dietary pattern with high intake of plant food, decreased obesity risk by providing an balanced n-6/n-3 PUFAs ratio.

## Introduction

Malnutrition is one of important international indicators for monitoring nutritional status and health in populations. Childhood overweight and obesity is becoming an emerging face of malnutrition. In 2020, there are now 38.3 million of children with overweight in global, an increase of estimated 21% since 2000 [1]. Childhood overweight and obesity have immediate and longer-term effects on children’s health including glycolipid metabolic abnormalities, diabetes, high blood pressure and adult obesity [2].

Obesity is predominantly attributed to unbalanced nutritional metabolism, especially for energy intake or expenditure imbalance [3]. Fatty acids (FAs) are major components of lipids, which provide an important energy source as nutrients. Fat metabolism and dietary fatty acids have been concerned to affect obesity [4]. Some epidemiological studies have examined the relationship between dietary FAs and obesity [5, 6]. However, there are inconclusive evidences regarding the associations between the amount and types of FAs intake and obesity [7]. The potential reasons of these inconsistencies may attribute to diet FAs can’t reflected endogenous metabolism because of the potential interactions of diversified foods FAs sources.

In recent year, dietary pattern analysis considering the potential interactions between foods intake and nutrients is widely used to explore to the associations of diet intake with nutritional diseases [8]. The relationship between dietary patterns and obesity of children has been a persistent concern in many previous studies [9, 10]. However, the methods of dietary patterns mainly depended on principal component analysis and index-based methods in previous studies, the etiology of diseases related to diet wasn’t elucidated. Reduced rank regression (RRR) can develop dietary patterns related to the given health outcomes by using disease-related information as response variables. These response variables respresented the pre-hypothetical intermediates between dietary pattern and diseases, can test a putative hypothesis of disease pathophysiology and is helpful for mechanistic evidence [11].

Blood FAs biomarkers represented dynamic change of individual metabolism, provides not only information related to FAs diet intake of an individual, but also information related to endogenous metabolism [12, 13]. Our previous study displayed that blood docosahexaenoic acid (DHA, C22:6n-3) as the predominant n-3 polyunsaturated fatty acids (PUFAs) was associated with diet intake and obesity in Chinese children [14]. Hence, FAs biomarkers as the response variables may be used to explore the diet related etiology of children’s obesity. However, the composition of fatty acid profiles were complex, and FAs with different structures have different biological functions. Our previous results failed to clarify the mechanism of dietary and related diseases with fatty acid metabolism. Therefore, in this study, we further developed FAs-related dietary patterns based on erythrocytes FAs profiles as response variables by RRR and then explored the associations of the FAs-related dietary patterns with overweight and obesity among Chinese children.

## Materials and methods

### Subjects

The study sample was drawn from an earlier cross-section survey of 435 children in a single primary school selected using a cluster sampling. This observational study was conducted on children aged 4 to 7 years old in Xiangtan, Hunan province, located in South Central China. Details of children are described in elsewhere [15]. In this study, 21 were excluded due to incomplete dietary assessment or not collect the blood sample.

### Fatty acids analysis

Venous whole blood samples were collected and centrifuged at 3,000 × g for 10 min to separate the erythrocytes and plasma. These samples were stored at − 80 ℃ after separation. For the analysis of erythrocyte FAs profiles, 50 µl erythrocyte after thawing and 200 µl internal standard working solution (IS) was firstly added in 10 ml glass vial. Then, 1 mL 3 N methanolic hydrochloric acid was added for transmethylation at 90 ℃ for 1.5 h in hermetic condition. After cooling the vials to room temperature, 2 mL hexane was added, and the vial was vortexed for 10 s. The upper (hexane) layer was transferred and evaporated to dryness under N_2_. Finally, the residue was dissolved in 1 mL hexane before the analysis. The samples (1 µL) were analyzed using a gas chromatography mass spectrometer (GCMS-QP2010, Shimadzu Corp., Kyoto, Japan) and separated using an HP-88 column (dimensions: 100 m × 0.25 mm × 0.20 μm; Agilent Technologies, Santa Clara, CA, USA). The SupelcoTM 37 Component FA methyl esters Mix (Sigma-Aldrich, St. Louis, MO, USA) was used to identify key FAs via chromatograms. Quantification was based on calibration with methyl nonadecanoate (Aladdin, Shanghai, China) as the IS. The detailed analysis procedure was reported in our previous study [16]. The results of erythrocyte FAs profiles were expressed in µg/mL.

### Overweight and obesity assessment

Weight (kg) and height (cm) were measured with an electronic height and weight measurement instrument. Body Mass Index (BMI) was calculated by weight (kg) dividing square of height (m). The evaluation criteria of overweight and obesity for children based on WHO Child Growth Standards (2006). Z score of BMI-for-age were calculated. Overweight was BMI for 1<age Z score ≦ 2 and Obesity was>2 [17]. Besides, children with overweight or obesity were included in group of overweight or obesity.

### Dietary pattern assessment

Food frequency questionnaire was used to collect the dietary intake information. The estimated portion size and frequency over the previous 12 months of each food item for each child’s diet intake was recorded by asking for their caregivers. The frequency was recorded in terms of times per day, week, month, or year; portion sizes were expressed in grams or milliliters. The mean daily intake of each food item was calculated using the estimated portion size and frequency. Total of 55 food items were recorded and further categorized into 19 food groups based on similarities in nutrient profiles or processing methods. The 19 food groups were rice, wheat floor, coarse cereals, tubers, soybean and its products, meat, poultry, egg, fish, shrimp, crab and shellfish, milk and its products, leafy vegetable, leaf-off vegetable, fresh beans, fungi and algae, fruits, beverage, nuts, and snacks. The details had been reported in our previou study [15].

FAs related dietary patterns were constructed using RRR analysis with the intakes of 19 food groups as independent variables and erythrocyte SFAs, MUFAs, n-3 PUFAs and n-6 PUFAs concentration as response variables, respctively. The RRR methods are conducted in the special procedure PLS of the SAS System for Windows, release 9.4 (SAS Institute, Inc.). The number of dietary patterns is equal to the number of selected responses for RRR. Factor loadings for independent variables represented the correlation coefficients between food items and dietary patterns. Factor loadings for response variables represented the correlation coefficients between erythrocyte FAs and dietary patterns. The variables with factor loadings >|0.2| were considered to contribute significantly to the dietary pattern. The factor score as the sum of the products of the factor loading coefficients and standardized intake of each food group associated with the pattern were calculated, which can be used in subsequent statistical analysis. A higher factor score represented children more preferred to this dietary pattern [18].

### Other related variables

Sociodemographic information was collected during the FFQ interview, and comprised children’s age, sex, caregiver’s group (parents or grandparents/others), occupation (unemployed, non-public institution, or public institution), education level (junior and below, senior, or college and above), and annual family economic income. The age was divided into two groups (4 to 5 years or 6 to 7 years). The annual family income was subdivided into below 20,000 yuan, 20,000 to 50,000 yuan, and 50,000 yuan group and above.

### Ethics approval

All participants provided written informed consent. The study protocol was approved by the Ethics Committee of the Hunan Provincial Center for Disease Control and Prevention (HNCDC-BJ20190003).

### Statistical analysis

Categorical variables were expressed as numbers and percentages. Continuous variables were expressed as median (M) and interquartile range (IQR) due to skewed data. The differences of overweight and obesity with normal children on demographic characteristics were determined by Chi-square test. Mann-Whitney Test was run to determine the differences of FAs profiles between normal and overweight and obesity children. The associations of FAs related dietary patterns with overweight and obesity among children were explored by logistic regression analysis. The factor score of FAs related dietary patterns as independent variables, and overweight, obesity, and overweight or obesity as dependent variables, were conducted, respectively. Model 1was unadjusted; model 2 was adjusted for age, sex, caregiver’s groups, caregiver’s occupation, education, family economic level. The odds ratio (OR) and 95% confidence interval (CI) were calculated to determine the strength of associations. *P* < 0.05 was considered to indicate statistical significance. Statistical analyses were performed using SPSS software (version 13.0; SPSS, Inc., Chicago, IL, USA).

## Results

### Demographic characteristics of children with overweight and obesity

Table 1 showed the demographic characteristics of children. A total of 414 children aged 4 to 7 years old participated in the study. The prevalence of children’s overweight, obesity and overweight or obesity were 6.52, 4.59, and 11.11%, respectively. Comparing to normal children, boy had higher prevalence of obesity than girl (*P* < 0.05). However, no significant differences on overweight and obesity children were found for children’s sex, caregiver’s group, occupation, education level, and annual family economic income, comparing to normal children.

**Table 1:** The demographic characteristics of Chinese children (*n* (%) with overweight and obesity

Demographic variables	*N*	Overweight	Obesity	Overweight or obesity
Gender				
Boy	217	15 (6.91)	15 (6.91)*	30 (13.82)
Girl	197	12 (6.09)	4 (2.03)	16 (8.12)
Age				
4 to 5 year	202	17 (8.42)	9 (4.46)	26 (12.87)
6 to 7 year	212	10 (4.72)	10 (4.72)	20 (9.43)
Caregiver				
Parents	238	14 (5.88)	12 (5.04)	26 (10.92)
Grandparents and others	176	13 (7.39)	7 (3.98)	20 (11.36)
Caregiver’s occupation				
Public institution staff	35	2 (5.71)	2 (5.71)	4 (11.43)
Non public institution staff	150	8 (5.33)	10 (6.67)	18 (12.00)
Unemployment	229	17 (7.42)	7 (3.06)	24 (10.48)
Caregiver’s education				
College and above	36	3 (8.33)	2 (5.56)	5 (13.89)
Senior	115	5 (4.35)	5 (4.35)	10 (8.70)
Junior and below	263	19 (7.22)	12 (4.56)	31 (11.79)
Family economic level				
50,000 and above	128	10 (7.81)	6 (4.69)	16 (12.50)
20,000 to 50,000	183	12 (6.56)	9 (4.92)	21 (11.48)
Below 20,000	103	5 (4.85)	4 (3.88)	9 (8.74)
Total	414	27 (6.52)	19 (4.59)	46 (11.11)

*:compare to normal group by Chi-square test, *P* < 0.05

### FAs profiles composition of children with overweight and obesity

Table 2 showed that twenty five types of FAs were detected in erythrocyte of children, including nine saturated fatty acids (SFAs), six monounsaturated fatty acids (MUFAs), six n-6 polyunsaturated fatty acids (PUFAs), and four n-3 PUFAs. The first four FAs for concentration in erythrocyte of normal children were C16:0 (190.92 (164.01, 216.21) µg/L), C18:0 (129.02 (108.26, 148.04) µg/L), C18:1n-9 (82.61 (67.81, 97.92) µg/L) and C18:2n-6 (81.67 (60.48, 98.03) µg/L). Furthermore, the concentration of C14:0 were significantly higher in children with overweight or obesity than normal (*P* < 0.05). Besides, comparing to normal, children of obesity had lower level of C14:1n-5 (*P* < 0.05) and C22:6n-3 (*P* < 0.05). However, no significant difference was found for other FAs on children with overweight and obesity, comparing to normal.

**Table 2:** The erythrocyte FAs concentration among children with overweight and obesity (M(IQR), µg/L)

FAs	Normal	Overweight	Obesity	Overweight or obesity
C14:0	3.94 (3.55, 4.49)	4.31 (3.73, 4.82)	4.30 (3.80, 4.98)	4.31 (3.76, 4.83)*
C15:0	1.70 (1.55, 1.92)	1.78 (1.54, 2.00)	1.70 (1.60, 1.88)	1.72 (1.58, 1.99)
C16:0	190.92 (164.01, 216.21)	190.02 (175.83, 226.02)	184.78 (152.48, 202.78)	189.92 (166.16, 212.00)
C17:0	2.38 (2.08, 2.74)	2.50 (2.16, 2.82)	2.34 (1.93, 2.52)	2.44 (2.05, 2.70)
C18:0	129.02 (108.26, 148.04)	137.32 (120.10, 150.09)	122.51 (103.85, 139.78)	132.18 (118.97, 146.17)
C20:0	4.11 (3.69, 4.60)	4.24 (3.82, 4.70)	3.96 (3.79, 4.34)	4.21 (3.80, 4.57)
C22:0	10.00 (8.21, 12.66)	10.84 (9.30, 13.78)	9.66 (7.14, 11.05)	10.38 (8.54, 12.49)
C23:0	0.00 (0.00, 0.00)	0.00 (0.00, 2.03)	0.00 (0.00, 0.00)	0.00 (0.00, 1.42)
C24:0	23.32 (17.76, 30.00)	24.56 (21.19, 32.45)	20.51 (16.89, 27.95)	23.32 (18.06, 29.07)
SFAs	366.90 (315.35, 422.16)	383.11(338.45, 429.32)	362.83 (299.85, 387.44)	366.01 (334.37, 410.85)
C14:1n-5	4.34 (0.00, 5.88)	5.20 (0.00, 6.43)	0.00 (0.00, 5.05)*	4.58 (0.00, 6.06)
C16:1n-7	1.73 (1.35, 2.29)	1.71 (1.38, 2.50)	1.78 (1.56, 2.87)	1.77 (1.49, 2.62)
C18:1n-9	82.61 (67.81, 97.92)	76.25 (67.78, 93.96)	79.13 (66.28, 91.44)	78.47 (67.50, 92.82)
C20:1n-9	2.65 (1.95, 3.18)	2.59 (2.11, 3.46)	2.43 (2.02, 2.96)	2.56 (2.09, 3.17)
C22:1n-9	4.29 (3.52, 4.87)	4.19 (3.46, 4.93)	4.53 (3.71, 4.89)	4.33 (3.61, 4.90)
C24:1n-9	25.30 (18.34, 31.08)	27.35 (20.67, 30.42)	24.97 (15.48, 28.92)	26.23 (19.41, 29.83)
MUFAs	120.15 (99.83, 142.46)	119.46 (108.14, 142.34)	121.80 (95.84, 138.00)	121.31 (101.01, 139.37)
C18:3n-3	1.80 (1.46, 2.07)	1.74 (1.16, 1.93)	1.95 (1.54, 2.50)	1.76 (1.29, 2.14)
C20:3n-3	0.00 (0.00, 0.00)	0.00 (0.00, 0.00)	0.00 (0.00, 0.00)	0.00 (0.00, 0.00)
C20:5n-3	1.54 (0.00, 2.07)	1.18 (0.00, 2.38)	1.68 (0.98, 2.22)	1.63 (0.00, 2.38)
C22:6n-3	14.16 (7.76, 19.06)	10.51 (4.10, 19.09)	11.00 (4.92, 13.78)*	10.76 (4.57, 18.58)
n-3PUFAs	17.75 (9.88, 23.10)	14.41 (6.51, 23.24)	14.22 (8.51, 18.86)	14.31 (7.39, 22.85)
C18:2n-6	81.67 (60.48, 98.03)	69.34 (45.20, 95.55)	84.39 (57.93, 90.79)	72.48 (50.78, 91.90)
C18:3n-6	0.00 (0.00, 0.36)	0.00 (0.00, 0.98)	0.00 (0.00, 1.40)	0.00 (0.00, 1.09)
C20:2n-6	2.59 (2.11, 3.01)	2.67 (1.77, 3.11)	2.31 (1.72, 2.95)	2.59 (1.75, 3.02)
C20:3n-6	6.79 (4.93, 8.51)	6.73 (3.55,8.94)	6.24 (4.14, 7.71)	6.45 (3.99, 8.93)
C20:4n-6	77.61 (46.93, 96.42)	67.11 (21.13, 97.60)	59.62 (33.48, 82.50)	67.07 (30.56, 93.76)
C22:2n-6	0.00 (0.00, 1.40)	0.00 (0.00, 0.00)	0.00 (0.00, 1.30)	0.00 (0.00, 0.00)
n-6PUFAs	173.59 (118.89, 203.37)	148.60 (82.95, 206.17)	154.81 (108.96, 196.26)	151.70 (84.92, 197.04)
PUFAs	189.81 (128.82, 227.03)	160.97 (88.28, 233.10)	172.26 (117.47, 211.83)	166.61 (94.08, 215.77)
Total FAs	671.64 (556.11, 775.03)	693.92 (571.82, 776.69)	620.62 (538.52, 730.35)	658.28 (556.63, 739.52)

*:compare to normal group by Mann-Whitney Test, *P* < 0.05

**Fig. 1:**
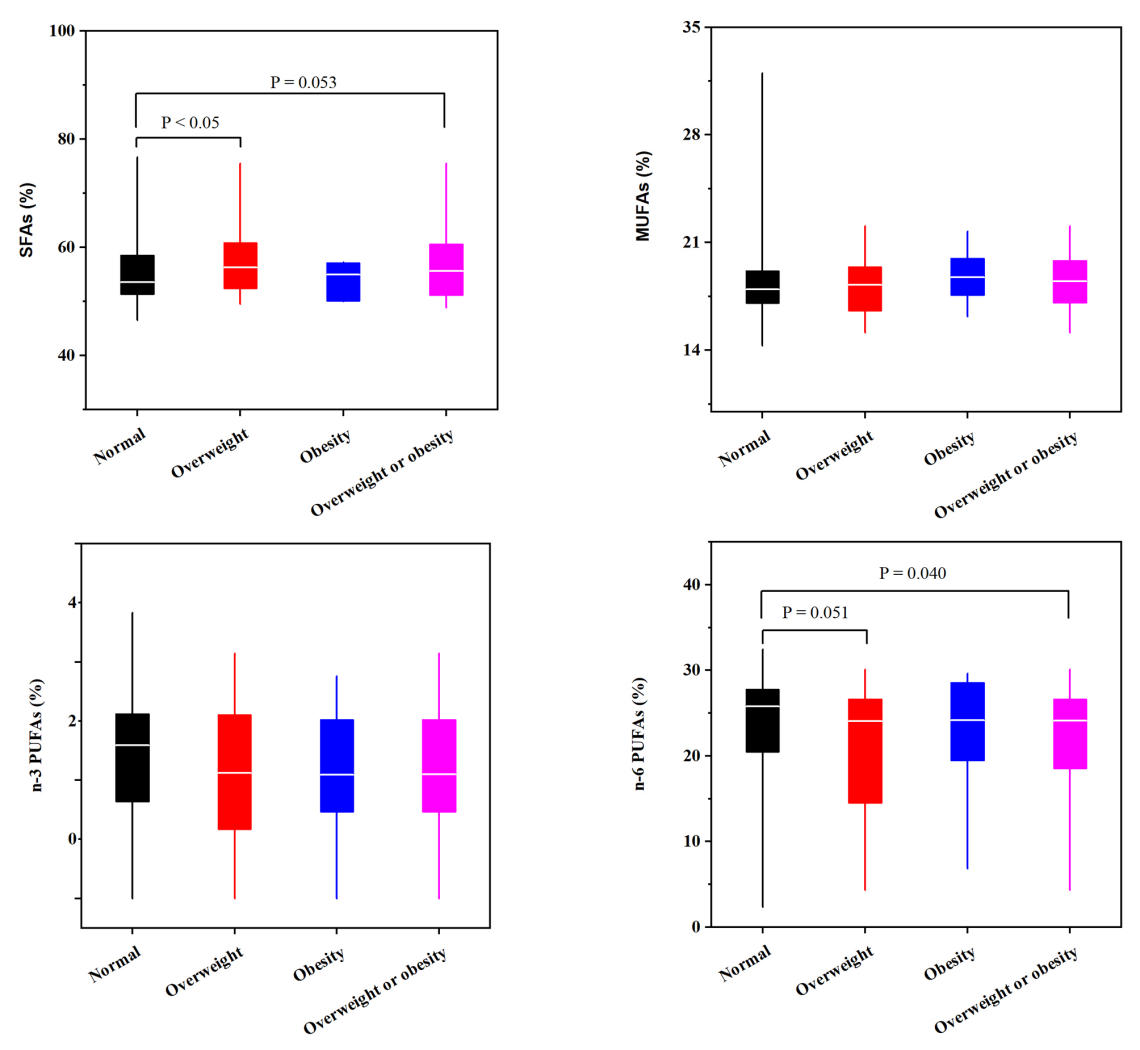
The erythrocyte FAs compositions among children with overweight and obesity. The differences of erythrocyte FAs compositions between normal and overweight, obesity, and overweight or obesity was conducted by Mann-Whitney Test

In addition, the relative compositions were caculated for SFAs, MUFAs, n-3 PUFAs and n-6 PUFAs, expressed as percentage (%). Comparing to normal, children with overweight (*P* < 0.05), and overweight or obesity (*P* ≈ 0.05) had higher proportion of SFAs, but lower proportion of n-6 PUFAs (Fig. 1). Accordingly, the concentrations of SFAs, MUFAs, n-3 PUFAs and n-6 PUFAs in erythrocyte of children were selected as response variables to conduct overweight and obesity related dietary patterns by RRR, respectively.

### FAs related dietary of children

**Fig. 2:**
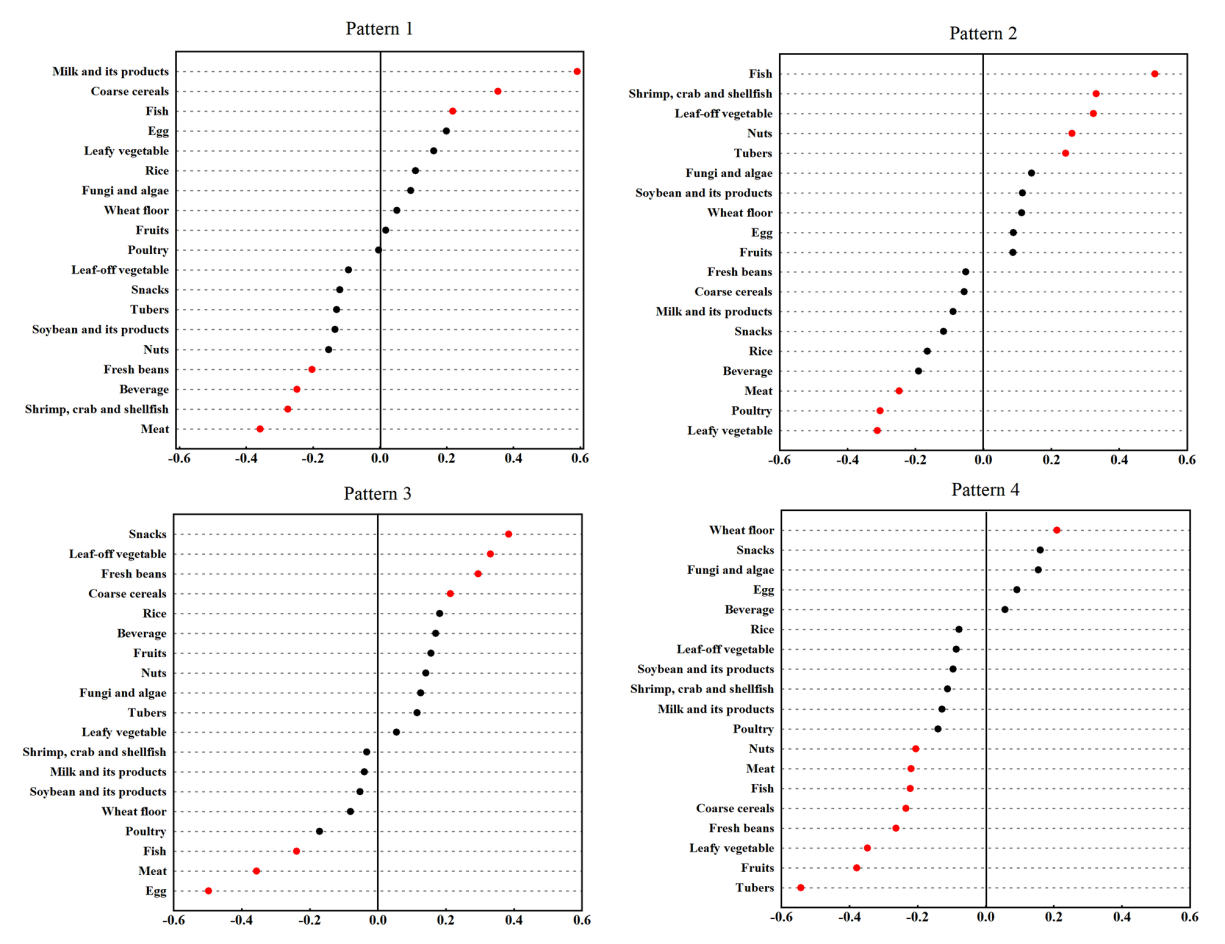
The factor loading of food items for FAs related dietary patterns by RRR

Four FAs related dietary patterns were identified explained 22.02% variation in the independent variables and 6.14% variation in the response variables by RRR. Pattern 1 was mainly characterized by a high consumption frequency of milk and its products, coarse cereals and fish, but low intake of meat, shrimp, crab and shellfish, beverage and fresh beans. Pattern 2 was positively correlated with the intake of fish, shrimp, crab and shellfish, leaf-off vegetable, nuts, and tubers, but negatively correlated with the intake of leafy vegetable, poultry and meat. Pattern 3 was associated with high intake of snacks, leaf-off vegetable, fresh beans, and coarse cereals, but low intake of egg, meat and fish. Pattern 4 had high positive loadings for wheat floor, but negative loadings for tubers, fruits, and leafy vegetable (Fig. 2).

**Fig. 3:**
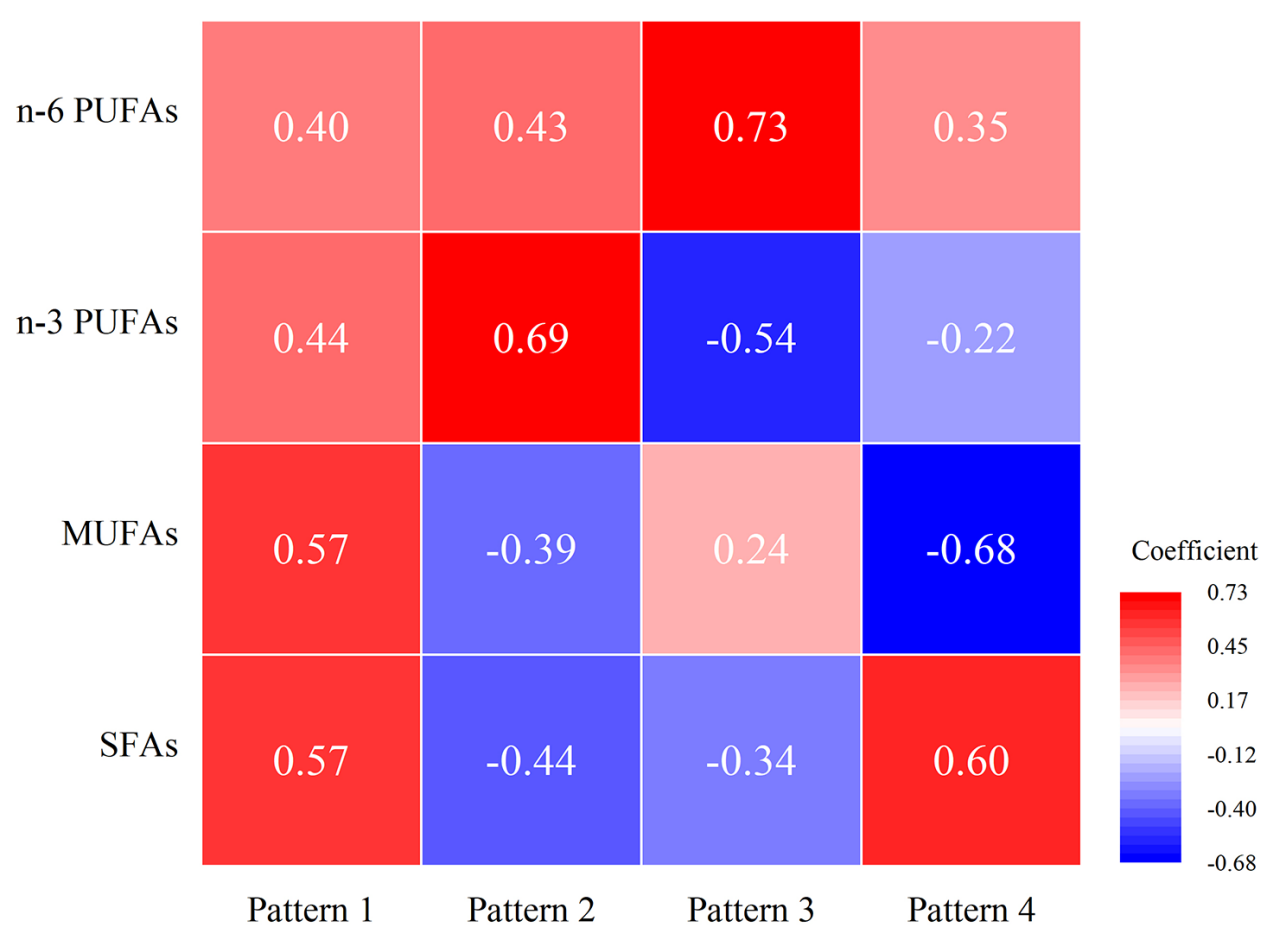
The response score coefficient for erythrocyte FAs on FAs related dietary patterns by RRR

Moreover, pattern 1 showed positively strong correlations with all FAs (*r* = 0.57 for SFAs, *r* = 0.57 for MUFAs, *r* = 0.44 for n-3 PUFAs, and *r* = 0.40 for n-6 PUFAs. Pattern 2 had positively correlations with n-3 PUFAs (*r* = 0.69), but negatively with SFAs (*r*=-0.44). Pattern 3 is positively strong associated with n-6 PUFAs (*r* = 0.73), but negatively strong with n-3 PUFAs (*r*=-0.54). Pattern 4 is positively strong associated with SFAs (*r* = 0.60), but negatively strong with MUFAs (*r*=-0.68) (Fig. 3).

### The association of FAs related dietary patterns with overwight and obesity

Table 3 showed the results of the associations of FAs related dietary patterns with overweight and obesity among children by logistic regression analysis. In the crude model 1, higher adherence to pattern 2 was associated with decreasing risks of children’s overweight (OR = 0.620, 95% CI:0.411 ∼ 0.936, *P* = 0.023); pattern 3 was associated with decreasing risks of children’s obesity (OR = 0.502, 95%CI:0.327 ∼ 0.769, *P* = 0.002). After multivariable adjustment, pattern 2 still have a significantly decreased overweight risk (OR = 0.580, 95%CI: 0.375 ∼ 0.895, *P* = 0.014), and pattern 3 have a significantly decreased obesity risk (OR = 0.518, 95%CI: 0.325 ∼ 0.827, *P* = 0.006).

**Table 3:** The associations of FAs related dietary patterns with overweight and obesity among children by logistic regression analysis

Dietary patterns	Overweight		Obesity		Overweight or obesity	
	OR(95%CI)	*P*	OR(95%CI)	*P*	OR(95%CI)	*P*
Pattern 1						
Model 1	0.953 (0.594, 1.529)	0.842	0.958 (0.557, 1.649)	0.878	0.957 (0.667, 1.372)	0.809
Model 2	0.897 (0.557, 1.446)	0.656	0.940 (0.549, 1.610)	0.822	0.923 (0.644, 1.324)	0.664
Pattern 2						
Model 1	0.620 (0.411, 0.936)	0.023	0.899 (0.555, 1.456)	0.665	0.727 (0.526, 1.005)	0.053
Model 2	0.580 (0.375, 0.895)	0.014	0.961 (0.592, 1.559)	0.872	0.729 (0.523, 1.017)	0.063
Pattern 3						
Model 1	1.049 (0.683, 1.611)	0.826	0.502 (0.327, 0.769)	0.002	0.735 (0.533, 1.012)	0.059
Model 2	1.038 (0.669, 1.610)	0.869	0.518 (0.325, 0.827)	0.006	0.755 (0.544, 1.048)	0.093
Pattern 4						
Model 1	0.982 (0.664, 1.453)	0.927	0.831 (0.544, 1.268)	0.390	0.911 (0.675, 1.229)	0.542
Model 2	1.042 (0.699, 1.553)	0.840	0.882 (0.549, 1.419)	0.605	0.973 (0.712, 1.332)	0.866

Model 1: unadjusting; Model 2: adjusting for age, gender, caregiver, caregiver’s occupation, education, and family economic level

## Discussion

In this study, the prevalence of overweight, obesity, and overweight or obesity were 6.52, 4.59, and 11.11% in Chinese children, respectively. Twenty five types of FAs were detected in erythrocyte of children and four FAs related dietary patterns were identified. The dietary pattern was positively correlated with n-3 PUFAs, but negatively with SFAs, which have a significantly decreased overweight risk. The pattern positively strong associated with n-6 PUFAs, but negatively strong with n-3 PUFAs, which have a significantly decreased obesity risk.

Childhood obesity is associated with serious health, which is recognized as “one of the most serious public health challenges” of this era. In this study, the prevalence of overweight or obesity was 11.11% in Chinese children age 4 to 7 years old, which was lower the level European children aged 2 to 7 years (17.9%) [19], but similar to the prevalence of children with 6 years old and below in China from the China Health and Nutrition Survey (11.5%) [20]. However, many studies reported childhood overweight and obesity have increased dramatically worldwide in recent decades [21]. In China, the prevalence of childhood overweight and obesity increased over 60% from 2005 to 2010 [22]. Therefore, the problem of overweight and obesity on Chinese children still should be focused.

Furtermore, this study first reported the dietary patterns related to FAs status by RRR. Four FAs related dietary patterns were identified in this study. Pattern 1 showed positively strong correlations with all FAs, characterized by a high intake milk and its products, coarse cereals and fish, similar to the traditional pattern reported in preschool children of Lebanons, which decreased risk of preschool overweight [10]. Pattern 2 positively related to n-3 PUFAs, but negatively to SFAs, had high intake of fish, shrimp, crab and shellfish, leaf-off vegetable, nuts, and tubers, similar to the Mediterranean diet [23]. Pattern 3 was positively strong associated with n-6 PUFAs, but negatively strong with n-3 PUFAs, characterized by high intake of snacks, leaf-off vegetable, fresh beans, and coarse cereals, but low intake of egg, meat and fish, similar to the plant pattern from the same population indentified by factor analysis in our previou study [15]. Pattern 4 had high positive loadings for wheat floor, but negative loadings for tubers, fruits, and leafy vegetable. No similar dietary pattern among Chinese children was found in provious studies [24–26].

Many studies had confirmed that dietary patterns are associated with overweight and obesity [27], but few studies clarified the etiology. Fat metabolism is strictly connected with obesity. The FAs profiles was found to have a key role in describing the scenario for the various phases of weight increase, from overweight to obesity [28]. In this study, no significant difference was found for majority of erythrocyte FAs concentration between normal and overweight or obesity of children. But we found comparing to normal, children with overweight, and overweight or obesity had higher proportion of SFAs, but lower proportion of n-6 PUFAs in erythrocyte. In addition, our results showed the dietary pattern 2 positively correlated with n-3 PUFAs, but negatively with SFAs, decreased overweight risk. This pattern eriched fish, shrimp, crab and shellfish, leaf-off vegetable, nuts, represented the natural sources of n-3 PUFAs. Several studies had reported SFAs increase the risk of overweight and obesity (especially for long-chain SFAs) [29], and high n-3 PUFAs decrease the risk [5]. Moreover, the dietary pattern 3 positively strong associated with n-6 PUFAs, but negatively strong with n-3 PUFAs, decreased obesity risk. n-6 PUFAs as highly prothrombotic and proinflammatory, increased cardiometabolic risk. A balanced n-6/n-3 PUFAs ratio is considered an important biomarker in the prevention and management of obesity [5]. The dietary pattern 3 had high intake of plant foods. Foods from plants contain a good balance of omega-6 and omega-3 fatty acids [5]. These results showed the dietary patterns by influencing FAs compositions play an important role in the development of overweight and obesity.

The major strengths of this study are that RRR was used to conduct dietary patterns by using erythrocyte FAs related to overweight and obesity as response variables, which may elucidate the etiology of overweight and obesity related to diet. Our results showed that FAs reflected metabolic distinctions from overweight to obesity, were closely linked to dietary patterns. The dietary pattern with high intake of aquatic products or plant food, may decrease the risk of overweight and obesity in children by regulating FAs metabolism. These results may be useful for designing more precise nutritional intervention for children’s overweight and obesity. Our study also has several limitations. First, this was a cross-section study with small samples that some aspects should be further assessed in larger populations. Moreover, errors in recall was unavoidable due to FFQ method to collect the diet data. Lastly, only one school was selected, which may have affected the generalizability of the findings.

## Conclusions

In this study, four FAs related dietary patterns were identified. The dietary pattern with high intake of fish, shrimp, crab and shellfish, leaf-off vegetable, nuts, and tubers, decreased overweight risk by increasing n-3 PUFAs, and decreasing SFAs. The dietary pattern with high intake of plant food, decreased obesity risk by providing an balanced n-6/n-3 PUFAs ratio. The association of dietary pattern with overweight and obesity among children may be attributed to FAs metabolism.

## Data Availability

No datasets were generated or analysed during the current study.
